# Using National and Local Extant Data to Characterize Environmental Exposures in the National Children’s Study: Queens County, New York

**DOI:** 10.1289/ehp.0900623

**Published:** 2009-06-15

**Authors:** Paul J. Lioy, Sastry S. Isukapalli, Leonardo Trasande, Lorna Thorpe, Michael Dellarco, Clifford Weisel, Panos G. Georgopoulos, Christopher Yung, Margot Brown, Philip J. Landrigan

**Affiliations:** 1 Environmental and Occupational Health Sciences Institute, University of Medicine and Dentistry, New Jersey–Robert Wood Johnson Medical School and Rutgers University, Piscataway, New Jersey, USA; 2 Mount Sinai School of Medicine, New York, New York, USA; 3 New York City Department of Health and Mental Hygiene, New York, New York, USA; 4 National Children’s Study, Eunice Kennedy Shriver National Institute of Child Health and Human Development, National Institutes of Health, Department of Health and Human Services, Bethesda, Maryland, USA

**Keywords:** children, environmental measurements, EXIS, exposure index, exposure information system, microinventories, National Children’s Study, national databases, NCS

## Abstract

**Objective:**

The National Children’s Study is a long-term epidemiologic study of 100,000 children from 105 locations across the United States. It will require information on a large number of environmental variables to address its core hypotheses. The resources available to collect actual home and personal exposure samples are limited, with most of the home sampling completed on periodic visits and the personal sampling generally limited to biomonitoring. To fill major data gaps, extant data will be required for each study location. The Queens Vanguard Center has examined the extent of those needs and the types of data that are generally and possibly locally available.

**Data:**

In this review we identify three levels of data—national, state and county—and local data and information sets (levels 1–3, respectively), each with different degrees of availability and completeness, that can be used as a starting point for the extant data collection in each study location over time. We present an example on the use of this tiered approach, to tailor the data needs for Queens County and to provide general guidance for application to other NCS locations.

**Conclusions:**

Preexisting and continually evolving databases are available for use in the NCS to characterize exposure. The three levels of data we identified will be used to test a method for developing exposure indices for segments and homes during the pilot phase of NCS, as outlined in this article.

The National Children’s Study (NCS) is a large-scale, longitudinal epidemiologic study of 100,000 children to be selected from 105 locations across the United States, authorized by Congress to identify human exposures and preventable causes of childhood disease (Landrigan et al. 2006; NCS 2007a). To assess residential exposures, researchers will collect dust, water, and other samples from homes and analyze biological specimens for chemical contamination as well as for other markers of exposure. These measurements alone cannot fully characterize environmental exposures of participants in this study; other data are needed for the testing of certain key hypotheses of the NCS, a point that was not a prominent concern during the initial design stage (Özkaynak et al. 2005). Thus, extant environmental data sets specific to study locations, counties, and segments will be needed to supplement the new study-specific measurements and provide supplementary study-related data at a relatively low cost. These data have the most value when they are used in conjunction with other resources for exposure characterizations, including computational source-to-dose modeling (e.g., Georgopoulos and Lioy 2006).

Queens, New York (USA), is one of seven national NCS pilot study locations. In this article we present an initial characterization of environmental databases and associated information available for Queens, where the objective is to recruit a representative sample of 1,250 live births. We analyzed national-, regional-, and county-level databases and other data-gathering efforts and engaged the local community leadership during the process. We describe the environmental characteristics of Queens in conjunction with the approach for conducting segment characterization and, eventually, home characterization and present this approach in generalized form for subsequent use in other NCS locations.

## Segment Selection in the NCS

The design of the NCS has been extensively described elsewhere, especially focusing on the multistage clustered sampling approach to enroll a sample of 100,000 live births representative of all American children (Branum et al. 2003; Landrigan et al. 2006; Trasande and Landrigan 2004; Trasande et al. 2006).

Queens, New York, the most ethnically diverse county in the United States (Kasinitz et al. 1998), was selected in 2005 as one of seven pilot study locations. Enrolled families will participate in a minimum of 14 data collection encounters: at least one visit before conception; twice during pregnancy; at birth; at 6, 12, and 18 months of age in early childhood; at 3, 5, 7, 9, and 12 years of age in childhood; and at 16 and 20 years of age. The population sampling strategy was designed to be nationally representative and to facilitate assessment of relevant community-level exposures.

NCS employed a three-stage sampling design; the primary sampling units (PSUs) are the 105 locations. Each PSU was divided into strata based on both geographic and demographic characteristics. Strata were created to maximize homogeneity and obtain roughly equal numbers of births per smaller sampling unit. Because of the difficulty of creating coherent segments in larger, more populous counties, an extra stage was introduced to increase operational efficiency and to reduce costs.

To meet the NCS-required objective that the sampling strategy reflect neighborhoods, community boundaries previously defined by the New York City (NYC) Department of City Planning (2007) were used to stratify Queens neighborhoods, based on administrative boundaries and demographic and historical information. Strata were checked by Queens Vanguard Center (QVC) researchers for homogeneity using census data, including information on race, ethnicity, education, income, and foreign-born status. Ultimately, 18 noncontiguous, relatively homogeneous, and equal-sized strata were created that would be the primary focus of environmental data collection.

The QVC researchers provided NCS planners with population size, birth counts from 2000 through 2004 (to estimate numbers of future births), and demographics by census block. Geographic unit boundaries constructed by the NCS Coordinating Center (Westat, Rockville, MD) were reviewed by QVC researchers to ensure that the geographic units did not cross the neighborhood boundaries defined by the NYC Department of City Planning. The final step of the sampling design was the creation of segments in the selected geographic units, with one segment selected from each geographic unit.

**Figure 1:**
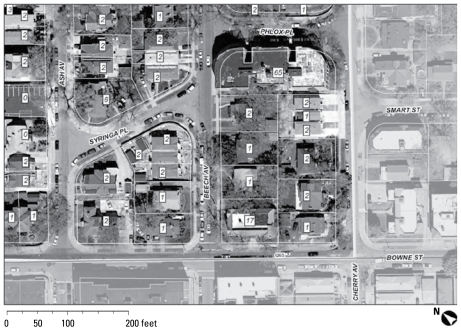
A close-up of a practice segment provides an aerial view with street names, lot lines, and an estimated number of dwelling units. Offices are designated by “o”; the numbers indicate number of dwelling units in a building.

Geographic information system (GIS) tools were used at all levels of the sampling design process in Queens: determining the boundaries of each segment, ensuring contiguity and compactness, and defining boundaries in relation to highways and railroads that can divide a neighborhood or otherwise result in differences in contextual exposures. Geocoded information was incorporated on the number of housing units; the distribution of single-family homes, row houses, and apartments within segments; lot and building outlines; and street names. Test mappings were developed, as shown in Figure 1. Actual segments were defined to be more contiguous in an attempt to maximize homogeneity, localize environmental exposures, and increase operational efficiency while maintaining sampling design requirements. Layering secondary information on maps, such as local bus routes and subway stops, zoning information, and nearby parks and landmarks, assisted field teams in recruiting participants.

## Environmental Data and Variables for Exposure Characterization within the NCS

**Figure 2:**
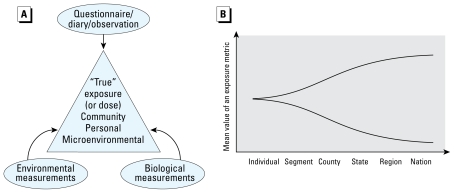
(*A*) The three components that are necessary to estimate “true” exposures or doses to selected subjects (based on the NCS Research Plan). (*B*) Increase in uncertainties when data at a coarse level are used to supplement segment- or individual-specific information for characterizing exposures. The upper and lower curves denote the upper and lower bounds of estimates of an exposure metric. Increasing difference indicates higher uncertainties.

Early childhood and maternal exposure to pollutants are important aspects of the NCS. The long-term goals of exposure characterization for the NCS are to understand and characterize “true” exposures using a combination of multimethod assessments and to use the metrics to test various NCS hypotheses. Figure 2A, adapted from the NCS Research Plan (NCS 2007c), shows three components that are necessary for estimating exposure or dose: environmental measurements, biological measurements, and questionnaires. These components are greatly aided by computational models for exposures and biological doses. Further, three levels of data are associated with exposure characterization and estimation within the scope of NCS: community-, household-, and individual-level data, dependent on time, locations, and activities. At each level, measurements need to be taken or values estimated for appropriate variables (NCS 2007b). Thus, for each child in the study, the collection of environmental data will be associated with “fields of influence,” for example, adjacent to the child, the household of the child, and the surrounding community. Data from all levels can be combined and used to establish the exposure or dose for each participating child.

For many persistent organic chemicals and some metals (i.e., chemicals with long biological half-lives), validated biomarkers will be used to determine the levels of persistent compounds in blood, urine, and the like, before, during, and after pregnancy (Barr et al. 2005). The biospecimen sample collection will switch from the mother to the child after birth. The results from biomarker measurements can indicate whether or not an exposure has occurred for this suite of compounds, but for most agents they cannot identify the source or route of exposure. Eventually, exposure reconstruction modeling tools may employ these data to test NCS hypotheses in conjunction with other exposure-related information (e.g., activity patterns, home measurements) (Georgopoulos et al. 2009). However, one must remember that exposure reconstruction is more feasible for chemicals with a long half-life, a condition that does not represent the full range of agents that can affect exposure–response relationships within NCS populations.

One component of the NCS that provides information to support environmental exposure characterizations will be the examination of the mother and child’s home using observation and questionnaires/diaries. Information will be collected at an initial home visit and updated periodically throughout the study. The observations will provide both qualitative and quantitative information and guidance on the types of sources or environmental insults that might actually be experienced by the children from contact with contaminant source emissions or from other specific types of contaminant contact related to their activities. The questionnaires and details on their potential use with biological and chemical markers of exposure are found in the NCS design document (NCS 2007b).

Actual environmental measurements in the home will include settled dust and wipe samples, specific water samples from a subset of homes, and possibly air samples within sub-studies. These data will be used to help estimate a child’s individual exposure over time. The current list of measurements to be completed in the households is summarized in the NCS Research Plan (NCS 2007a) and includes multiple compounds, multiple media, and multiple exposure pathways [see Supplemental Material, Table S1 (doi:10.1289/ehp.0900623.S1 via http://dx.doi.org)].

For the communitywide environmental measurements, a number of plausible measurement techniques can be implemented during the NCS. However, given the complexity of the study and the fact that resources needed to conduct environmental field measurements are quite extensive, the NCS has determined that most environmental data must be retrieved primarily from available databases.

The cost and the human resources needed to establish new community monitoring networks within the NCS data collection plan, which would include each segment and aim to characterize all contaminants of importance over 2 decades, are considerable, and the associated effort would be daunting. Thus, a coherent set of local ambient environmental data for Queens is not expected to be available to test all study hypotheses using the monitoring data [see Supplemental Material, Table S1 (doi:10.1289/ehp.0900623.S1)]. Short-term sampling will be implemented and will provide critical information on contaminant and exposure variability within Queens and the other NCS locations. These data alone, however, will not provide a comprehensive environmental and microenvironmental characterization for cumulative exposure estimates of each NCS participant. Therefore, it is necessary to identify the overall multiexposure route processes based upon a segment’s location and the proximity of outdoor sources to the individual households, as well as upon the presence of indoor sources.

Characterizing the impact of local environmental factors requires thoughtful and careful application of national, regional, and local data (Figure 2A), which need to be linked in a consistent manner, even though different data collection approaches and protocols may have been used for each medium, pollutant, or scale. For example, when national-level data sets are used to estimate exposures at an individual or segment level, the uncertainties associated with application of such data are high, as shown in Figure 2B. Having an understanding of local demographic, geographic, and contextual information about neighborhoods is critical in order to adapt or for adopting national sampling plans to reflect local circumstances.

The QVC has developed an approach for obtaining the information and identifying environmental databases that can be used to establish baseline or background levels in the county for specific pollutants and media; this approach will eventually be available for use in comprehensive models for exposure–dose characterization within the segments (Georgopoulos and Lioy 2006). The above databases would provide a path forward for use of data collected by long-term measurement programs available from a variety of organizations. The goal would be to incorporate the data into long-term characterizations of exposures for the participating children.

## Levels of Data Available to NCS Locations

**Figure 3:**
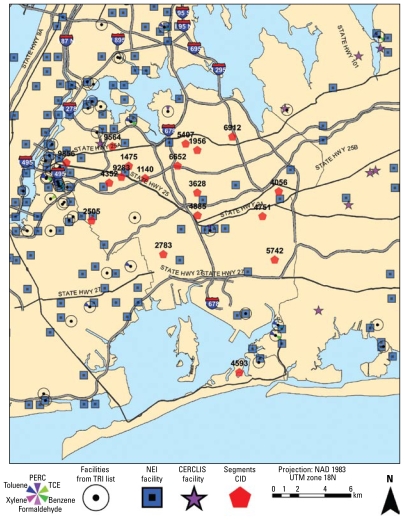
Overview of the Queens County study area and the segments (arbitrary number) selected in the NCS. National Emissions Inventory (NEI) and the U.S. EPA’s Comprehensive Environmental Response, Compensation, and Liability Information System (CERCLIS) facilities are also shown (highlighting the different degrees of proximity to facilities that influence environmental characteristics among different segments), as well as facilities reporting emissions of volatile organics [data from U.S. EPA’s 2005 Toxics Release Inventory (TRI)]. Abbreviations: CID, centroid and ID; NAD, North American datum; PERC, perchloroethylene; TCE, trichloroethylene; UTM, Universal Transverse Mercator coordinate system.

The data for a typical NCS location can be broadly classified as being of three types: national databases, state and local databases, and segment-scale data from detailed observations. To provide a focus for the following sections, Figure 3 presents a map of the borough of Queens, New York, that shows highways, roadways, and secondary tributaries, the 18 segments (identified by random numbers and no actual boundaries for the purposes of this article) selected for recruitment, and locations of specific sources. The segments reflect a cross-section of the diversity in population, activities, industry, and commercial services that currently exist in Queens. Each recruitment segment has unique demographic and environmental characteristics; as NCS begins selecting birth mothers and their unborn babies for participation, it is necessary to identify and understand the salient characteristics of each segment, including the environment.

## Data Sources

The identification of environmental data applicable for Queens starts with “level 1” databases to establish the NCS location background or baseline concentrations. In most cases these can be derived from well-established national programs. These will also be available to many other NCS locations. The “level 2” data represent a finer resolution, typically based on within-county data sources, and can come from either national or state programs. Assembling data of this level requires “mining” databases that exist within federal, state, county, and city governments to define pollution sources as well as issues of concern based upon major accidents, spills, and the like, within the community. The data, in various forms, should also be accessible to other NCS locations. The “level 3” information is also essential for each location segment and takes into account a multitude of possible community sources. Level 3 data require walk-through surveys of the community (and of potential pollution sources) to provide a much finer level of relevant information for each segment and location. Knowledge of local pollution that affects a community is of value to provide an initial characterization of both the segments and the homes for environmental conditions. The walk-through needs to be completed for each individual county segment to assist in problem definition.

**Figure 4:**
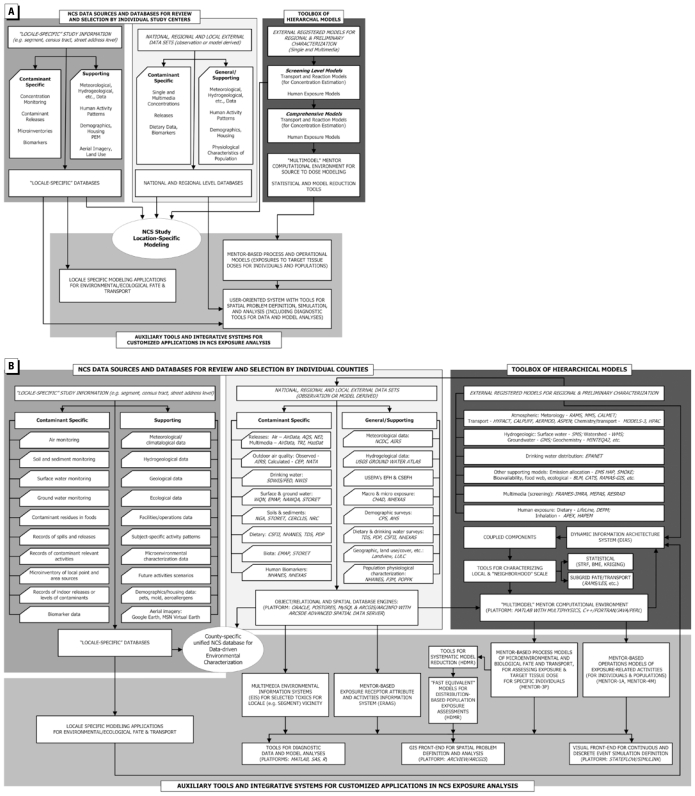
A prototype EXIS framework for the NCS: a summary overview (*A*) and a more detailed overview (*B*), including specific databases, models, and diagnostic tools to be employed. Figure adapted and expanded from Georgopoulos et al. (2005). Abbreviations: AERMOD, AMS/EPA Regulatory Model; AHS, American Housing Survey; AIRS, Aerometric Information Retrieval System; APEX, Air Pollution Exposure Model; ASPEN, Assessment System for Population Exposure Nationwide; BLM, Biotic Ligand Model; BME, Bayesian Maximum Entropy; CALPUFF, CALifornia PUFF model; CATS, Contaminants in Aquatic and Terrestrial ecosystems; CEP, Cumulative Exposure Project; CHAD, Consolidated Human Activity Database; CPS, Current Population Survey; CSEFH, Child-Specific Exposure Factors Handbook; DEPM, Dietary Exposure Potential Model; DIAS, Dynamic Information Architecture System; EFH, Exposure Factors Handbook; EMAP, Environmental Monitoring and Assessment Program; EMS-HAP, Exposure Modeling System-Hazardous Air Pollutants; EPANET, Environmental Protection Agency water NETwork model; FRAMES-3MRA, Framework for Risk Analysis in Multimedia, Multipathway, Multireceptor Risk Assessment; GMS, Groundwater Modeling System; HAPEM, Hazardous Air Pollutant Exposure Model; HPAC, Hazard Prediction and Assessment Capability; HYPACT, Hybrid Particle And Concentration Transport Model; LULC, Land Use and Land Cover; MEPAS, Multimedia Environmental Pollutant Assessment System; MINTEQA2, Metal Speciation for Equilibrium for Surface and Ground Water; NAWQA, National Water Quality Assessment; NCDC, National Climatic Data Center; NGA, National Geochemical Atlas; NHEXAS, National Human Exposure Assessment Survey; NWIS, National Water Information System; P3M, Physiological Parameters for Population Based Pharmacokinetic Modeling; PEM, Personal Exposure Modeling; POPPK, Population Pharmacokinetics; RAMAS-GIS, Risk Analysis and Management Alternatives Software-Geographical Information Systems; RAMS/LES, Regional Atmospheric Modeling System/Large Eddy Simulation; RESRAD, RESidual RADiation; SMOKE, Sparse Matrix Operator Kernel Emissions; SMS, Surface Water Modeling System; STRF, Spatio-Temporal Random Field; WMS, Watershed Modeling System; WQN, Water Quality Network.

An exposure index (EI) is under development for use in the NCS and will be calculated for each segment and eventually each home in Queens and another pilot location. Such an index will be a starting point for the classification of the 105 counties and their segments according to important environmental variables and potential community exposures. Figure 4 presents a schematic of the overall procedure (adapted from Georgopoulos et al. 2005). Figure 4A provides a summary overview of the levels of data that may be available for individual NCS locations, and how they would feed into the development of an EI. Figure 4B provides a detailed depiction of the databases and the types of models that can be used to develop an EI for each segment and each location. The goal is to develop a general methodology that can be applied across the entire set of NCS locations.

Examination of the present approach to acquisition of level 1–3 data shows that the data selected for the EI can have both near- and long-term applications. The prototype EI is currently under development for Queens and a rural NCS location and will be using an exposure information system (EXIS) for acquiring and assembling data for analysis (Georgopoulos and Lioy 2006). Details on the utility and application of an EXIS can be found in Georgopoulos et al. (2005, 2006).

## National Databases

Many national databases should be evaluated for use as level 1 extant data for an NCS location. Based on environmental media that have contaminants affecting Queens County, the following selections were made for assembling many types of information on this community. Other databases will probably be identified and accessed as the NCS becomes thoroughly involved with recruitment within all locations and segments across the nation.

### Air

Concentrations of many airborne pollutants across the United States are available from AirData, a computer-based repository that provides access to both atmospheric concentrations, maintained in the Air Quality System (AQS), and atmospheric emissions, available in the National Emissions Inventory (NEI) [U.S. Environmental Protection Agency (EPA) 2007a]. The AQS contains information that is assembled in a consistent manner from all ongoing monitoring programs nationwide to meet regulatory requirements. AirData contains summaries and details of air monitoring data for the current and several prior years, the latest available estimates of air pollutant emissions from major sources, the overall regulatory compliance status of those sources, and agency contact information (U.S. EPA 2007a). The NEI provides emission inventories that include information on stationary sources and on mobile sources, both on-road and off-road. This information is updated periodically, most recently in 2005 (U.S. EPA 2008c).

All these data pertain to the criteria pollutants and air toxics: carbon monoxide, nitrogen dioxide, sulfur dioxide, ozone, particulate matter, and lead. The AirData Web site permits generation of GIS layers and maps at scales ranging from the entire United States to a single county, showing locations of air monitoring sites and major point sources as well as county total emissions (U.S. EPA 2007b). Thus, the NCS can take advantage of a rather sophisticated ambient network to review and decide the applicability to an individual county, such as Queens, based upon locations and measured pollutants. However, the data available would be sparser in smaller or less densely populated areas.

### Water

The Safe Drinking Water Information System, federal version (SDWIS/FED), is a U.S. EPA database storing basic information about the nation’s drinking water supply (U.S. EPA 2008b). This information comes from the states and U.S. EPA’s regional offices and is reported for every public water system. SDWIS/FED stores the information that the U.S. EPA needs to monitor from approximately 175,000 public water systems. The database includes the name of the public water system, information about the type of area served by the water system (e.g., households, schools, restaurants, gas stations, or rest areas), number of people served by the water system, operating season (year-round or seasonal), regulator of the water system, when (or if) a water system has violated any national drinking water standard, and what (if any) follow-up actions have been taken to make sure the water system returns to compliance after a violation. Access to SDWIS/FED information can be obtained through the U.S. EPA Web site Envirofacts (U.S. EPA 2008a), which makes a subset of SDWIS/FED information easily available to anyone with Internet access.

The U.S. EPA Storage and Retrieval (STORET) database (U.S. EPA 2009c) is an Oracle-based database used for the storage of biological, chemical, and physical data for public water systems. The national database, administered by U.S. EPA, covers all states, territories, and jurisdictions of the United States along with bordering areas of Mexico and Canada. STORET was developed in 1964 as a collection and reporting system for water quality sampling, observation, and measurement activities that occur in these systems and comprehensive descriptors of the event during which samples were collected or the measurements performed.

The National Water Quality Assessment program was established by the U.S. Geological Survey (USGS) in 1991 (USGS 2008). The program systematically collects chemical, biological, and physical water quality data from rivers and streams, as well as ground-water and drinking water across the United States and British Columbia. Included are the concentrations of metals in groundwater, surface water/bed sediment, and mixture of surface water and groundwater. Through the Water Quality Network (USGS 2000), the USGS has systematically monitored streams in watersheds for the past 30 years from 679 sites throughout the United States for two national stream water quality networks: the Hydrologic Benchmark Network and the National Stream Quality Accounting Network. Each network provides national and regional descriptions of stream water quality conditions and trends for 63 physical, chemical, and biological properties using relatively consistent sampling and analytical methods. It also includes information about water quality and stream flow station attributes including drainage area, latitude, longitude, and so on. However, there is no standard for either the frequency of collection or the reporting of temporal data, which may be monthly, yearly, or even daily for intensive surveys. Consistency of reporting is a barrier to the use of these data in a longitudinal context, as needed for the NCS.

### Food

Food consumption data for the U.S. population are collected by the U.S. Department of Agriculture (USDA) Agricultural Research Service (ARS, SR Release 21) using the Continuing Survey of Food Intake by Individuals (CSFII) (Centers for Disease Control and Prevention 2008; Dwyer et al. 2003; USDA 2008b). CSFII examines a nationally representative sample of individuals of all ages, who were asked to provide food intakes on 2 nonconsecutive days, along with socioeconomic and health-related information. More than 1,000 variables are collected on household and individual nutrition intake, food groups, health/disease status, diet, health knowledge, and demographics.

The Total Diet Study (TDS), a market basket study, has been conducted continuously by the U.S. Food and Drug Administration since 1961 to characterize the U.S. population’s dietary habits (U.S. Food and Drug Administration 2005). The TDS database provides baseline information on the levels of nutrients and contaminants in foods, including radionuclides, pesticide residues, and toxic and nutritional elements. The TDS “shopping list” is developed taking into consideration compatibility with National Health and Nutrition Examination Survey (NHANES) (Centers for Disease Control and Prevention 2008), which contains data from dietary recall interviews and food questionnaires. The market basket studies are done in various cities in the western, north central, southern, and northeastern United States. The Pesticide Data Program (PDP) (USDA 2009) is a national program focusing on pesticide residues in agricultural commodities in the U.S. food supply, with an emphasis on commodities highly consumed by infants and children. In the absence of subject-specific dietary intake data, data from CSFII, TDS, and PDP can be coupled to approximate chemical exposures from dietary sources.

### Multimedia contaminants

The Toxics Release Inventory (TRI) contains information on annual estimates of allowed emissions of specific toxic chemical releases and other waste management activities reported by many industries (U.S. EPA 2008d). Data summaries are available for *a*) fugitive air, *b*) stack air, *c*) total air emissions, *d*) surface water discharge, *e*) underground discharge, *f*) releases to land, and *g*) total releases. Facilities are self-reporting, and the data may be derived from monitoring information or estimation with quality assurance performed by the U.S. EPA. Variations among facilities can result from the use of different estimation methodologies. In addition, limited and very general information is provided on chemical storage.

Trace metal levels in soil, sediment, and water samples collected throughout the United States between 1964 and 1995 are included in the National Geochemical Atlas database (USGS 2007), derived from a subset of the National Uranium Resource Evaluation and the Hydrogeochemical and Stream Sediment Reconnaissance data (USGS 2006).

### Waste sites

Information on the release of 180 hazardous substances from Superfund sites or from emergency events, and the effects of hazardous substances on the health of human populations are presented in the Hazardous Substance Release and Health Effects Database (HazDat), the scientific and administrative database of the Agency for Toxic Substance and Disease Registry (ATSDR 2008; Fay and Mumtaz 1996). HazDat also contains data from U.S. EPA’s Comprehensive Environmental Response, Compensation, and Liability Information System (CERCLIS) database, including site CERCLIS number, site description, latitude/longitude, operable units, and additional site information (U.S. EPA 2009a). Included are sites on the National Priority List; the information provided includes the names and classes of contaminants and the studies or activities being performed at the sites. The data in these files are updated regularly, in some cases weekly.

Additional data sets include the following:

- National Land Cover Data (Multi-resolution Land Characteristics Consortium 2008), a 21-class land cover classification scheme applied consistently over the United States.
- Topologically Integrated Geographic Encoding and Referencing data [U.S. Census Bureau (USCB) 2005], a database of geographic features (e.g., roads and rivers).
- Land Use and Land Cover (USGS 009).
- State Soil Geographic Database for soil properties, relevant to potential contaminations in the future (USDA 2008a).

The National Oceanographic and Atmospheric Administration (NOAA) National Climatic Data Center (NCDC) provides local meteorologic data that can be coupled with emissions data and dispersion modeling to estimate local, neighborhood-scale levels of some air pollutants (NOAA 2005). The USCB provides LandView and MARPLOT software products to aid in viewing U.S. EPA, USCB, and USGS data and maps (USCB 2009).

## Representative Queens County Multilevel Environmental Databases

Representative examples of the ongoing acquisition of national-, county-, and segment-level data (defined as levels 1, 2, and 3, respectively) for Queens County are provided in the following sections. These web sources are meant to illustrate data availability and utility in the NCS. The evaluation identified heterogeneity in the environmental information that could be encountered as the NCS evolves in Queens. The list of data sources examined specifically for Queens County is listed below; these do not include food analysis because the design and needs will be evaluated after receiving information collected from questionnaires. Because extant databases will be periodically queried and the corresponding data retrieved and stored, the NCS will automatically address changes in the databases. In addition, aggregated data sets will be provided a version number, and major changes between versions will be explicitly reported in the NCS.

### Air: emissions data sources

- U.S. EPA National Emissions Inventory (http://www.epa.gov/ttn/chief/trends)
- U.S. EPA Toxics Release Inventory (http://www.epa.gov/tri)

### Air: modeled concentration and risk estimates

- U.S. EPA National-Scale Air Toxics Assessment (http://www.epa.gov/ttn/atw/nata2002)

### Air: monitored concentrations

- U.S. EPA AirData (http://www.epa.gov/oar/data)

### Water

- NYC Department of Environmental Protection Well Data (http://nyc.gov/html/dep/html/drinking_water/groundwater.shtml#wells)
- New York State drinking water summary (http://www.health.state.ny.us/environmental/water/drinking/facts_figures.htm)
- U.S. EPA information on violations of water quality (http://oaspub.epa.gov/enviro/sdw_form_v2.create_page?state_abbr=NY)

### Soil

- ATSDR HazDat (http://permanent.access.gpo.gov/lps21/hazdat.html)
- National Response Center (NRC) chemical spills (http://www.nrc.uscg.mil/download.html)
- New York State Department of Environmental Conservation (NYSDEC) Spills Incident Database (http://www.dec.ny.gov/cfmx/extapps/derexternal/index.cfm?pageid=2)
- NYSDEC site remediation information (http://www.dec.ny.gov/cfmx/extapps/derexternal/index.cfm?pageid=3)
- USGS National Geochemical Atlas (http://tin.er.usgs.gov/geochem)

### Integrative

- U.S. EPA Envirofacts Warehouse (http://oaspub.epa.gov/enviro/ef_home2.land)

#### Level 1

The above-listed data sources identify major chemicals or indicator chemicals that may be emitted and may be present, if not persistent, in Queens County. Each of the databases is useful in developing criteria for selecting the types of information that can be relied on for long-term evaluation of exposure to the children born in each of the 18 segments in Queens. Further, these data will help identify any major information gaps based on sources or particular demographic or other types of variables that may lead to higher or lower exposure patterns among the children and their mothers. Unfortunately, the available databases are not consistent for contaminants across every exposure pathway—air, water, soil, and food—because the data were not designed to be aggregated under one database. The networks associated with various agencies meet either certain regulatory requirements or risk management strategies to help understand the potential source–receptor relationships for emissions and to determine the degree of compliance to community regulations. For example, volatile species emitted into the air may or may not be persistent in the environment; therefore, some of these species will not be found in the water and soil. This does present a problem because there could be a bias in one of the three media toward data collected for persistent compounds or nonpersistent compounds when applied to test hypotheses. The issue will need to be evaluated for exposure bias when testing NCS hypotheses. The first indications of data utility and relevance will be identified during the development of the EI (Figure 4).

Multiroute and multipathway models will need to be employed to estimate the actual contribution to exposure from media other than those for which there are high levels or reportable levels of a particular compound (e.g., for drinking water, some compounds only have violations reported, but not the level of contamination needed to estimate total exposures or body burden). Many drinking-water systems report concentrations of the trihalomethanes, haloacetic acids (two main groups of disinfection by-products), metals, chlorine levels, nitrite, nitrates, and the like, as means, and the actual data for individual samples may be available on request from the U.S. EPA or local water companies.

#### Level 2

Figure 2A illustrates differences in exposures assessed using biomarkers and using personal-, household-, or community-level extant data desired by the NCS. Community pollution concentrations help to define the background or baseline conditions for particular media and for a particular segment in the NCS location. Therefore, one starts with data acquisition at the “grossest level” in the community (level 1) and then tailors the analyses of the data to the segments (level 2). At a minimum, these can be used to rank the sources and pollutants in segments and provide an index for potential “high-end” or “low-end” exposure. Clearly, the databases may be the same as listed in the preceding section, but the level of scrutiny increasing as one examines data for individual segments.

#### Level 3

A “walk(drive)-through” has been used in previous community environmental studies and is beneficial for identifying sources that normally are not regulated for emissions or the emissions are not easily quantified in state databases because many emission inventories are collected only at the county level (Lioy and Daisey 1987). However, the level 1 data should provide guidance in developing objective measures for level 3 characterization. Two classic examples of emission sources not individually characterized by many states are gas stations and dry cleaners. Both emit air toxics, and both are found in residential communities. Residential communities that have dry cleaners may need to consider whether families live above these establishments. Some of these facilities could be recruited for the present study and may have significant exposure to tetrachloroethylene (Schreiber et al. 1993). Similar approaches can be used to identify contaminated water and soil from state data sets.

Air concentrations of some pollutants can have a countywide distribution by virtue of how they are emitted or formed in the atmosphere. For level 3, a template was developed to identify potential emission sources for the walk/drive-through of each segment (Table 1). For Queens, a microinventory of 15 categories for grouping commercial and industrial sources was created (Table 1) to facilitate the characterization of the 18 segments. The information for each column was filled in after a drive/walk-through of each segment. The completed chart identified distinct industrial/commercial differences among segments. From the standpoint of ambient pollution levels, knowledge of these differences is important for identifying major district “hot spots” that may be associated with exposures of children to be recruited in the study. The initial microinventory should be updated periodically in Queens and at other NCS locations, during household visits, to provide a basis for characterizing changes in long-term exposure. In addition, a walk-through is planned by the NCS at approximately every 5 years, because major changes in environmental conditions in each segment can occur over that period of time.

### Representative illustrations of data available for the 18 NCS segments in Queens

Many of the currently available environmental databases for Queens can be incorporated within a GIS system to provide two types of information. The first is a systematic presentation of the location of major emission sources and the different pollutants of direct concern, as well as other types of pollutants or pollution classes. The second is a display of concentrations for individual pollutants estimated from models, or the actual concentrations that were measured at various fixed monitor locations or through sampling studies. Because of the diversity in population and sources of contamination within the 18 segments in Queens County, it is essential to understand location and segment-related contributions to exposure.

For purposes of illustration, we present two levels of GIS mapping for characterization of Queens. Figure 3 shows the locations of industrial sources in Queens County that release volatile organics from the TRI (U.S. EPA 2008d) and industrial emission sources from the NEI (U.S. EPA 2008c). It is clear that these sources are not evenly distributed throughout Queens and that they vary by types of emitted contaminant. Also shown are all the CERCLIS sites in Queens, which can be augmented with information on the types of pollutants present at each location; this provides a means of defining the distance between segments and site locations and allows evaluation of completed exposure pathways.

In addition to the emissions identified from the national databases, it was necessary to augment these data with information obtained at the state and local levels, including historical records. For example, the former Adams Brush Manufacturing Site is currently the location of Architecture HS 800C, a NYC public school. The soil at the site has been contaminated with semivolatile organic compounds, volatile organic compounds (VOCs), and other contaminants associated with historic anthropogenic fill materials and manufacturing activities. On site were two underground storage tanks, which were closed in place. The groundwater sampling conducted in 1999 revealed VOCs (tetrachloroethylene and trichloroethylene) at concentrations up to 1,300 ppb. Subsequent sampling in 2000 and 2002 showed significantly lower groundwater contaminant concentrations (22 ppb) (NYSDEC 2009a).

For many air toxics of importance, mobile emissions from gasoline-powered automobiles and motorcycles and from diesel engines in trucks and cars can have a major impact on exposure near and within residences. Major roadways for mobile source emissions are illustrated in Figure 3. In addition to stationary sources, the existing roadway information on number of vehicle-miles is coupled with mobile sources in the county. New modeling approaches can bring emissions characterization down to the scale of major and minor roadways. Obviously, to complete the quantitative estimates of air pollution, meteorologic data will need to be acquired for the NCS location and segments from the NOAA-NCDC or from state or local studies and then “scaled down” to the neighborhood level in order to become appropriate for air pollution exposure modeling.

**Figure 5:**
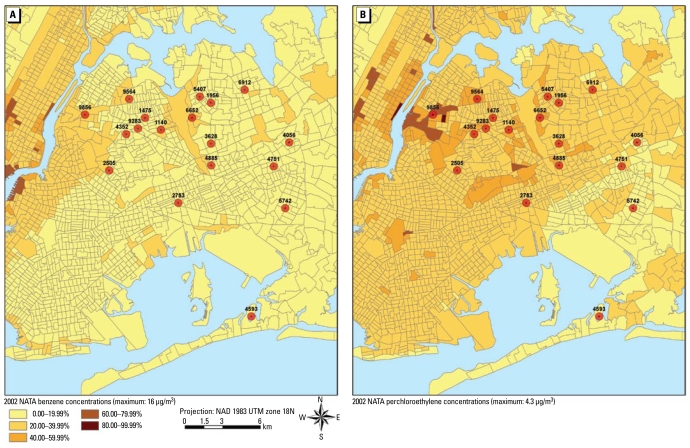
Estimates of annual average airborne concentration of benzene (*A*) and perchloroethylene (*B*) in Queens and surrounding counties. These estimates are based on U.S. EPA’s 2002 NATA (U.S. EPA 2009b) and can be used to provide “baseline” background concentrations.

The benefits of obtaining the emission data for stationary and mobile sources are 2-fold: the general qualitative characterization of the segments, and the use in the quantitative estimation of the exposure in each district using dispersion modeling and exposure characterization modeling. For extant data evaluation, air pollution exposure estimates originating from outdoor sources were obtained from the National-Scale Air Toxics Assessment (NATA) for 2002 (U.S. EPA 2009b). The U.S. EPA estimated concentrations of various air toxics using the Assessment System for Population Exposure Nationwide model and then estimated risk based on these estimated concentrations/exposures. As a screening tool, NATA can have a high degree of uncertainty in individual exposure estimation because it does not include variations in the emissions or the profile of human activities within the community. However, NATA does provide a baseline that can be updated on receipt of new information in NEI and related databases. Examples of the NATA estimates across Queens for benzene and perchloroethylene are shown in Figure 5.

Queens is served by the NYC water supply, which is primarily a citywide distribution system that has its sources northwest of NYC. Thus, in contrast to other cities and towns, the water is not from an urban surface water source or from local wells, and any problems would be identified directly from the citywide testing system. Few violations have been identified for pollutants of concern in Queens, although seasonal variations in the levels of disinfection by-products exist for the system. In addition, metal levels in water at the home level, particularly lead, can be influenced by the plumbing within each home, and exposures from water would need to be considered when interpreting the measured biomarker levels. The levels of contaminants in water are not captured in county- or citywide databases directly, but may be inferred from information on when the structure was built and when renovations to the plumbing occurred, to determine the types of piping, fitting, or solder that may exist. These types of data would need to be combined with questionnaire responses regarding use of tap water and filtration.

## Microinventory Report of Exposures for Community Engagement and Other Purposes

Detailed characterizations have been performed for each of the 18 segments of Queens County. Table 2 provides examples of what can be learned about local conditions for level 2 and level 3 databases for two Queens segments, 4593 and 9856 (no identified boundaries and a random numbering scheme). These segments were selected because each represents a very distinct type of location with respect to local environmental sources and concentrations.

Segment 4593 is located in the southern portion of Queens. Only a limited number of sites are listed in the CERCLIS, TRI, or NEI databases as being near segment 4593, indicating that major industrial sources probably have limited impact on the segment. The NATA risk analysis shows few compounds of concern above the median level within Queens. Thus, the risks and the concentrations of most air toxics were not above the average values for Queens, suggesting that the general exposures to air toxics in segment 4593 reflect NYC’s background levels. Other potential sources of contamination that have been reported include spills along the shore (dumping or leaking vessels) and emissions from JFK Airport. For example, during 2006–2007, seven spill incidents were reported (NYSDEC 2009b). In most cases the nature of the spill was oil, with the amounts of the larger spill reported in the database (NYSDEC 2009a).

The microinventory completed for segment 4593 provides information that is consistent with the above, confirming the presence of few major local sources that were not accounted for in the national inventories; only one gasoline station was located in the area, and no major commercial establishments that release substantial amounts of pollutants.

**Figure 6:**
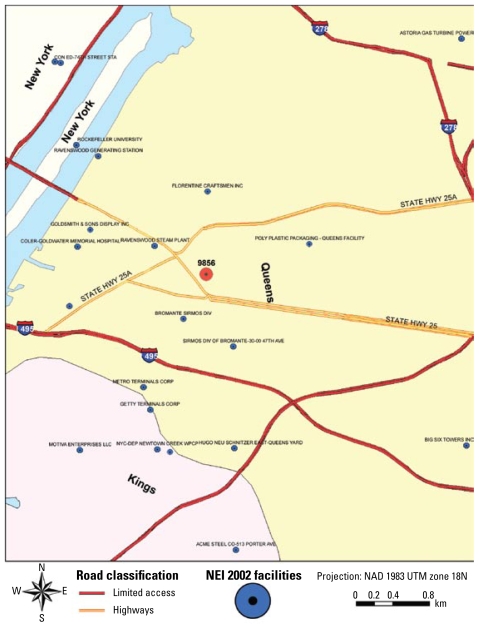
Facilities in the NEI 2002 within and nearby segment 9856 of the NCS in Queens, New York. (Boundaries are not provided for the segment.)

Segment 9856 is located in the northwestern portion of Queens County. In contrast to segment 4593, segment 9856 has a number of air pollution sources located within and immediately adjacent to the segment (Figure 6). There is an air quality monitoring site located just across the East River to the west in Manhattan. This can provide representative estimates of ambient levels for regional pollutants but still will not capture the local concentrations of many air pollutants that would reflect emissions from sources of air toxics within or adjacent to segment 9856. This will be a major issue because there are > 200 sources of multiple toxics located in and around segment 9856 (Table 2). The information is also consistent with the NATA estimates for the area, which indicate the presence of multiple air toxics that yield risks that are above the median value for Queens. Many of these sources could also contribute to soil or water pollution due to spills and runoff. A more detailed analysis of these data and other related information will be required vis-à-vis the locations of the subjects within the segment before one can complete an initial exposure characterization of the Queens population. For these cases, it is clear that calculation of segment and location EIs can yield large differences.

## Discussion and Conclusions

From the ongoing identification of extant data sources, it is clear that exposure-to-dose modeling will be essential for developing exposure profiles for each subject in the Queens, New York, NCS location. Identifying the appropriate types of extant data and information will also be of value in many other study locations beyond Queens to help test appropriate NCS hypotheses. The primary reason is that the number of environmental samples collected from individual homes will be insufficient to characterize family and child community exposures. Further, for the foreseeable future, personal exposure monitoring is prohibitively expensive to use in a longitudinal study designed to test the NCS hypotheses.

Each NCS location must consider its own demographics, sources, and overall activities and focus data gathering efforts for levels 1–3 so as to understand the potential environmental impacts to the children enrolled in the study. The data collected or identified for segments 4593 and 9856 show that each segment has “individualized” environmental characteristics; indeed, segment 4593 is predominantly residential, and there are only a few commercial facilities and some adjacent sites with emissions, whereas segment 9856 is a heavily commercial/industrial area in which residences are interspersed. Other segments in Queens are similar to segment 4593 or have commercial sources that place them, from the EI perspective, between segments 4593 and 9856. Such differences and similarities must be part of the EI to be determined for each Queens segment, and the approach should be tested for use in other NCS locations and to determine the utility in testing NCS hypotheses. Similarly, segments in other NCS locations must be evaluated to define and understand the source characteristics. These will assist in describing impact on the mothers and children residing there by constructing individualized exposure profiles.

The environmental releases in each segment and county will change over the course of the 20-year study; therefore, the NCS must also reevaluate the character and demography of the individual segments periodically to assess changes in the environmental quality of the area and in potential exposures of the study participants. For example, from the Harvard Six Cities study, the characteristics of the industrialized city of Steubenville, Ohio, were found to change dramatically from its inception of the study through a retrospective follow-up (Laden et al. 2006). Significant reductions in exposure to environmental contaminants in Steubenville led to improvements in the health of the local population. Changes in the characteristics of the NCS location segments, either positive or negative, must be accounted for in the examination of the NCS children’s health status. This continual reevaluation will need to occur periodically over the next 20 years.

**Figure 7:**
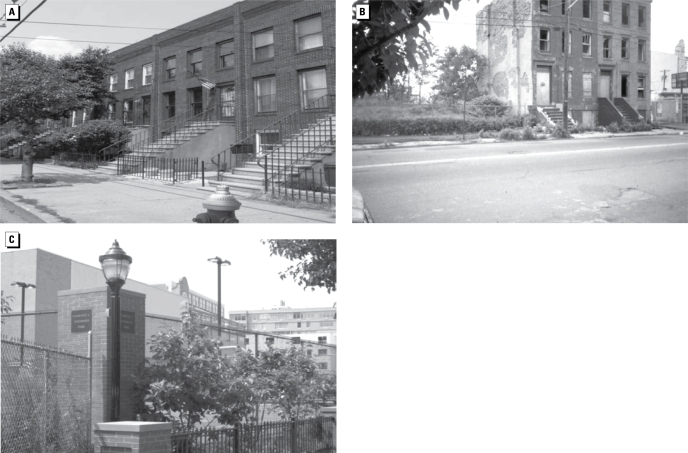
Grand Street in Jersey City, New Jersey. (*A*) Residential condominiums constructed pre-1991. (*B*) Unremediated chromium site across the street in 1991. (*C* ) Park at same location as former chromium site shown in (*B*) in July 2008.

It is also expected that over the 20-year period new pollutants and monitoring systems are likely to be established, and each should be evaluated for inclusion in the NCS database to test various hypotheses. An example of how exposures can change with time because of development of urban areas can be seen from a longitudinal study conducted in Jersey City, New Jersey. More than 25 years ago, many sections of Jersey City were blighted with residential chromium waste (Lioy et al. 1992). Today, the waterfront in Jersey City has been rebuilt, all known residential sites have been cleaned up, and there are now major efforts in place or planned to clean up the largest industrial sites laden with chromium (e.g., the Roosevelt Avenue drive-in theater site). Thus, the character of a major part of the community has changed and will continue to change, and exposures will continue to be reduced in the remediated areas. Figure 7 provides an example of the changes that occurred across the street from a condominium complex built in the late 1980s. In 1991, the location was blighted with chromium-contaminated soils and an abandoned building. The site now contains a park (Figure 7C), and there is also rapid development of the area adjacent to the Jersey City waterfront, a block away. Thus, the NCS has to be vigilant to document changes that occur over time in the Queens segments and the other segments in the selected counties.

There are concerns that need to be weighed regarding the use of extant data as a substitute for environmental samples directly collected from homes, schools, and child care centers. Use of extant data could result in bias toward data collected in a single or in multiple media for either persistent or nonpersistent compounds. Thus, multiroute and multipathway exposure and dose models will need to be employed to estimate the actual contributions to exposure of participants from various media, including where data are sparse or where data are provided only when a standard is violated for specific compounds. Each of the above requires multiple analysis steps and various types of modeling. Because these will take years to evolve, the concept of developing an EI has been put forward as part of the NCS to establish baselines for each segment and location. The EI will require development of simple but flexible algorithms to make it useful across segments within Queens, and the procedure should be tested elsewhere in the NCS study after its performance has been evaluated for Queens.

## Correction

In the manuscript originally published online, the U.S. EPA NATA data were from 1999; these data have been updated in text and Figure 5 to 2002.

## Figures and Tables

**Table 1:** Examples of the commercial and industrial microinventory approach for the 18 selected sampling segments in Queens, New York (preliminary data).

Segment ID	Commercial and industrial source: facility type (code)													
	Industrial site (I)	Gas station (G)	Mechanic shop (M)	Auto body shop (A)	Dry cleaner (D)	Laundromat (L)	Restaurant (R)	Bakery (B)	Printing center (P)	Woodworking shop (K)	Hair/nail salon (S)	Hospital (H)	Fire station (F)	Other (O)
4593		1				1	1	1				1	1	2 bus depots 1 landfill
5742		1	4		2		4				4		1	1 truck sales and repair 1 car wash
4751							1				2			
6912		1				1	2	1			2			
6652							1							
3628					1		1							
4885														
1140			1		1	2	11	4			3			
9856		24	16	10		1	30	6	8		1			Multiple (details not included)
2505				1		3	2							
1956					1		2				3	1		2 car parking lots
4352	1		5		1	1	1				1			1 warehouse with lifting machinery
2783		1			1		3							
5407					1		13	1			4			1 car parking lot
4056					2		3				1			
9564			1				2				1			
9283		2	14				6		2					
1475														

**Table 2:** Comparison of segments 4593 and 9856 for industrial and commercial enterprises that are likely sources of air emissions leading to exposure to the residents of those segments.

	No. of commercial or industrial sites^a^ with air emissions sources	
Enterprise	Segment 4593	Segment 9856
Warehouse/retail	1	42
Gas station	1	24
Mechanic shop		15
Auto body shop		11
Dry cleaner		1
Laundromat		1
Restaurant	1	30
Bakery		6
Printing center		8
Facility/store with large parking lot		34
Hair salon		1
Car dealership		12
Hospital	1	1
Fire station	1	
Car wash		5
Bus depot	2	1
Other		8 Commercial facilities
No. of sites in national database that are required to report air emissions in or near segment		
CERCLIS	2	2
TRI Inventory	4	11
NEI Inventory	6	30

a Sites were identified during walk/drive-through

## References

[b1-ehp-117-1494] ATSDR (Agency for Toxic Substance and Disease Registry) (2008). Hazardous Substance Release and Health Effects Database.

[b2-ehp-117-1494] Barr DB, Wang RY, Needham LL (2005). Biologic monitoring of exposure to environmental chemicals throughout the life stages: requirements and issues for consideration for the National Children’s Study. Environ Health Perspect.

[b3-ehp-117-1494] Centers for Disease Control and Prevention (2008). National Health and Nutrition Examination Survey.

[b4-ehp-117-1494] Dwyer J, Picciano MF, Raiten DJ (2003). Future directions for the integrated CSFII-NHANES: what we eat in America—NHANES. J Nutr.

[b5-ehp-117-1494] Fay RM, Mumtaz MM (1996). Development of a priority list of chemical mixtures occurring at 1188 hazardous waste sites, using the HazDat database. Food Chem Toxicol.

[b6-ehp-117-1494] Georgopoulos PG, Bandi S, Efstathiou C, Li W, Shade P, Tan H (2006). Infrastructure for an Arsenic Exposure Information System. Technical Report CERM.2006:01.

[b7-ehp-117-1494] Georgopoulos PG, Lioy PJ (2006). From theoretical aspects of human exposure and dose assessment to computational model implementation: the Modeling Environment for Total Risk studies (MENTOR). J Toxicol Environ Health B Crit Rev.

[b8-ehp-117-1494] Georgopoulos PG, Sasso AF, Isukapalli SS, Lioy PJ, Vallero DA, Okino M (2009). Reconstructing population exposures to environmental chemicals from biomarkers: challenges and opportunities. J Expo Sci Environ Epidemiol.

[b9-ehp-117-1494] Georgopoulos PG, Wang SW, Yang YC, Xue J, Zartarian V, McCurdy T (2005). Assessing Multimedia/Multipathway Exposures to Arsenic Using a Mechanistic Source-to-Dose Modeling Framework. Case Studies Employing MENTOR/SHEDS-4M.

[b10-ehp-117-1494] Kasinitz P, Bazzi M, Doane R (1998). Jackson Heights, New York. Cityscape J Policy Dev Res.

[b11-ehp-117-1494] Laden F, Schwartz J, Speizer FE, Dockery DW (2006). Reduction in fine particulate air pollution and mortality: extended follow-up of the Harvard Six Cities study. Am J Resp Crit Care Med.

[b12-ehp-117-1494] Landrigan PJ, Trasande L, Thorpe LE, Gwynn C, Lioy PJ, D’Alton ME (2006). The National Children’s Study: a 21-year prospective study of 100,000 American children. Pediatrics.

[b13-ehp-117-1494] Lioy PJ, Daisey JM (1987). Toxic Air Pollution: A Comprehensive Study of Non-criteria Air Pollutants.

[b14-ehp-117-1494] Lioy PJ, Freeman NC, Wainman T, Stern AH, Boesch R, Howell T (1992). Microenvironmental analysis of residential exposure to chromium-laden wastes in and around New Jersey homes. Risk Anal.

[b15-ehp-117-1494] Multi-resolution Land Characteristics Consortium (2008). National Land Cover Database.

[b16-ehp-117-1494] National Children’s Study Interagency Coordinating Committee (2003). The National Children’s Study of environmental effects on child health and development. Environ Health Perspect.

[b17-ehp-117-1494] NCS (2007a). The National Children’s Study Research Plan. Appendix H: Detailed Overview of Environmental Measures.

[b18-ehp-117-1494] NCS (2007b). The National Children’s Study Research Plan. Study Design.

[b19-ehp-117-1494] NCS (2007c). The National Children’s Study Research Plan. Rationale for Exposure Measures.

[b20-ehp-117-1494] NOAA (National Oceanographic and Atmospheric Administration) (2005). National Climatic Data Center.

[b21-ehp-117-1494] NYC Department of City Planning (2007). Projected Population Change by Neighborhood, New York City, 2000–2010.

[b22-ehp-117-1494] NYSDEC (2009a). NYSDEC Environmental Site Remediation Database.

[b23-ehp-117-1494] NYSDEC (2009b). NYSDEC Spills Incident Database.

[b24-ehp-117-1494] Özkaynak H, Whyatt RM, Needham LL, Akland G, Quackenboss J (2005). Exposure assessment implications for the design and implementation of the National Children’s Study. Environ Health Perspect.

[b25-ehp-117-1494] Schreiber JS, House S, Prohonic E, Smead G, Hudson C, Styk M (1993). An investigation of indoor air contamination in residences above dry cleaners. Risk Anal.

[b26-ehp-117-1494] Trasande L, Cronk CE, Leuthner SR, Hewitt JB, Durkin MS, McElroy JA (2006). The National Children’s Study and the children of Wisconsin. WMJ.

[b27-ehp-117-1494] Trasande L, Landrigan PJ (2004). The National Children’s Study: a critical national investment. Environ Health Perspect.

[b28-ehp-117-1494] USCB (U.S. Census Bureau) (2005). Topologically Integrated Geographic Encoding and Referencing (TIGER) System.

[b29-ehp-117-1494] USCB (U.S. Census Bureau) (2009). LandView 6.

[b30-ehp-117-1494] USDA (U.S. Department of Agriculture) (2008a). US General Soil Map (STATSGO2).

[b31-ehp-117-1494] USDA (U.S. Department of Agriculture) (2008b). What We Eat in America, NHANES.

[b32-ehp-117-1494] USDA (U.S. Department of Agriculture) (2009). USDA Agricultural Marketing Service’s Pesticide Data Program.

[b33-ehp-117-1494] U.S. EPA (U.S. Environmental Protection Agency) (2005). Toxic Release Inventory Program.

[b34-ehp-117-1494] U.S. EPA (U.S. Environmental Protection Agency) (2007a). AirData: Access to Air Pollution Data.

[b35-ehp-117-1494] U.S. EPA (U.S. Environmental Protection Agency) (2007b). AirData: Generating Maps and Reports.

[b36-ehp-117-1494] U.S. EPA (U.S. Environmental Protection Agency) (2008a). Envirofacts.

[b37-ehp-117-1494] U.S. EPA (U.S. Environmental Protection Agency) (2008b). Drinking Water Data & Databases.

[b38-ehp-117-1494] U.S. EPA (U.S. Environmental Protection Agency) (2008c). National Emissions Inventory (NEI) Air Pollutant Emissions Trends Data.

[b39-ehp-117-1494] U.S. EPA (U.S. Environmental Protection Agency) (2008d). Toxics Release Inventory (TRI) Program.

[b40-ehp-117-1494] U.S. EPA (U.S. Environmental Protection Agency) (2009a). Comprehensive Environmental Response, Compensation, and Liability Information System (CERCLIS) Database.

[b41-ehp-117-1494] U.S. EPA (U.S. Environmental Protection Agency) (2009b). Technology Transfer Network 2002 National-Scale Air Toxics Assessment.

[b42-ehp-117-1494] U.S. EPA (U.S. Environmental Protection Agency) (2009c). STORET.

[b43-ehp-117-1494] U.S. Food and Drug Administration (2005). Total Diet Study.

[b44-ehp-117-1494] USGS (U.S. Geological Survey) (2000). Data from Selected US Geological Survey National Stream Water-Quality Monitoring Networks (WQN) on CD-ROM.

[b45-ehp-117-1494] USGS (U.S. Geological Survey) (2006). National Geochemical Database: Reformatted Data from the National Uranium Resource Evaluation (NURE) Hydrogeochemical and Stream Sediment Reconnaissance (HSSR) Program.

[b46-ehp-117-1494] USGS (U.S. Geological Survey) (2007). National Geochemical Atlas: The Geochemical Landscape of the Conterminous United States Derived from Stream Sediment and Other Solid Sample Media Analyzed by the National Uranium Resource Evaluation (NURE) Program.

[b47-ehp-117-1494] USGS (U.S. Geological Survey) (2008). National Water Quality Assessment (NAWQA) Program.

[b48-ehp-117-1494] USGS (U.S. Geological Survey) (2009). Land Use and Land Cover (LULC).

