# Erratum

**Published:** 2010-05

In the article “Using National and Local Extant Data to Characterize Environmental Exposures in the National Children’s Study: Queens County, New York” by Lioy et al. [ Environ Health Perspect 117:1494–1504 (2009)], Shahnaz Alimokhtari was inadvertently omitted as an author. The corrected author names and affiliations are listed below.

***Paul J. Lioy,^1^ Sastry S. Isukapalli,^1^ Leonardo Trasande,^2^ Lorna Thorpe,^3^ Michael Dellarco,^4^ Clifford Weisel,^1^ Panos G. Georgopoulos,^1^ Christopher Yung,^1^ Shahnaz Alimokhtari,^1^ Margot Brown,^4^ and Philip J. Landrigan^2^***

^1^Environmental and Occupational Health Sciences Institute, University of Medicine and Dentistry, New Jersey–Robert Wood Johnson Medical School and Rutgers University, Piscataway, New Jersey, USA; ^2^Mount Sinai School of Medicine, New York, New York, USA; ^3^New York City Department of Health and Mental Hygiene, New York, New York, USA; ^4^National Children’s Study, Eunice Kennedy Shriver National Institute of Child Health and Human Development, National Institutes of Health, Department of Health and Human Services, Bethesda, Maryland, USA

The authors apologize for the error.

